# Correlates of STI testing among vocational school students in the Netherlands

**DOI:** 10.1186/1471-2458-10-725

**Published:** 2010-11-24

**Authors:** Mireille EG Wolfers, Gerjo Kok, Johan P Mackenbach, Onno de Zwart

**Affiliations:** 1 Municipal Public Health Service, Rotterdam Area, Infectious Disease Control Division, P.O. Box 70032, 3000 LP, Rotterdam, the Netherlands; 2 Department of Public Health, Erasmus University Medical Centre, P.O. Box 2040, 3000 CA Rotterdam, the Netherlands; 3 Department of Work and Social Psychology, Faculty of Psychology and Neuroscience, Maastricht University, P.O. Box 616, 6200 MD Maastricht, the Netherlands

## Abstract

**Background:**

Adolescents are at risk for acquiring sexually transmitted infections (STIs). However, test rates among adolescents in the Netherlands are low and effective interventions that encourage STI testing are scarce. Adolescents who attend vocational schools are particularly at risk for STI. The purpose of this study is to inform the development of motivational health promotion messages by identifying the psychosocial correlates of STI testing intention among adolescents with sexual experience attending vocational schools.

**Methods:**

This study was conducted among 501 students attending vocational schools aged 16 to 25 years (mean 18.3 years ± 2.1). Data were collected via a web-based survey exploring relationships, sexual behavior and STI testing behavior. Items measuring the psychosocial correlates of testing were derived from Fishbein's Integrative Model. Data were subjected to multiple regression analyses.

**Results:**

Students reported substantial sexual risk behavior and low intention to participate in STI testing. The model explained 39% of intention to engage in STI testing. The most important predictor was attitude. Perceived norms, perceived susceptibility and test site characteristics were also significant predictors.

**Conclusions:**

The present study provides important and relevant empirical input for the development of health promotion interventions aimed at motivating adolescents at vocational schools in the Netherlands to participate in STI testing. Health promotion interventions developed for this group should aim to change attitudes, address social norms and increase personal risk perception for STI while also promoting the accessibility of testing facilities.

## Background

Adolescents are at risk for acquiring sexually transmitted infections (STIs). In fact, worldwide, the prevalence of STIs is substantially greater in young adults than in many other populations [1]. Surveillance systems in various industrialized countries show that Chlamydia is the most commonly reported STI and that the highest prevalence of Chlamydia is observed among female adolescents [1,2].

In 2008, national surveillance figures for the Netherlands showed a concentration of Chlamydia cases in heterosexual young people under 25 years of age with low to moderately educated adolescents living in urban centers being most at risk. Additionally, STI prevalence was found to be higher among adolescents of non-Dutch ethnicity such as Antillean and Surinamese adolescents [2,3]. Because high risk immigrant adolescents do engage in sexual intercourse with their Dutch peers, this increases the likelihood of STI transmission between high and low prevalence groups. This kind of disassortative mixing, previously described in a Dutch study of high-risk migrant populations in the Netherlands, is most likely to occur among second generation and younger migrants [4]. Also, a nationwide cross-sectional study of sexual behavior among people aged 12 to 25 showed that participants with lower educational attainment exhibited more sexual risk behavior and less knowledge regarding STI and reproductive/contraceptive issues than their better educated counterparts [5].

Because effective prevention of STI relies on early case detection and treatment [6], the Netherlands offers free and anonymous testing facilities to populations at risk such as young people under the age of 25. Unfortunately, test rates among young heterosexual people remain low [5]. Interventions that promote STI testing among this risk group are thus imperative. Such interventions are particularly necessary among young adults attending vocational schools in urban areas. These students, generally aged 16 to 20, tend to be of non-Dutch ethnicity and are at risk for STI not only because of their lower level of education but also because they tend to have earlier sexual debuts and engage in more unsafe sex than Dutch-born adolescents, thus leading to more opportunities for STI exposure [5].

However, effective interventions that encourage STI testing among these students are severely limited, and no interventions have specifically targeted vocational students. Consequently, the Municipal Public Health Service (MPHS) in Rotterdam developed an intervention that targeted students attending vocational schools. This intervention was developed using the Intervention Mapping (IM) protocol, which proposes a stepwise approach to the theory and evidence-based development and implementation of interventions [7].

According to IM and to other health promotion planning models such as the Precede-Proceed Model [8], an important part in the first phase of intervention development (the needs assessment) is establishing important and changeable behavioral determinants of the target behavior. Globally, most studies on the determinants of testing behavior focus on populations at risk for HIV such as men who have sex with men (MSM) [9-17] or on injection drug users (IDUs) [15,18]. Although research on the determinants of HIV testing among MSM can be used to hypothesize the behavioral mechanisms of testing in heterosexual adolescents, the context of and social environment in which sexual risk taking among adolescents takes place is very different than it is among MSM. Also, the incidence as well as the perceived severity and consequences of infection differ substantially between HIV and other STIs affecting heterosexual adolescents in Western countries. Other studies have investigated which demographic variables and risk-taking behaviors are specifically related to STI testing among adolescents [19-22] but none have explored the behavioral determinants of STI testing among young heterosexual adults with a low educational attainment in the Netherlands. Clearly, there is a paucity of research on the psychosocial determinants of STI testing among this group. Studies that employ a comprehensive theoretical framework are also scarce. Of the few studies that have employed a theoretical framework, some have used a stage model to assess readiness for STI testing [21-23], some have employed the Health Belief Model [24-26], and yet others have studied single psychological constructs such as shame and stigma associated with STI testing [27], social support related to STI and healthcare utilization [28] and attitudes [29].

**Figure 1:**
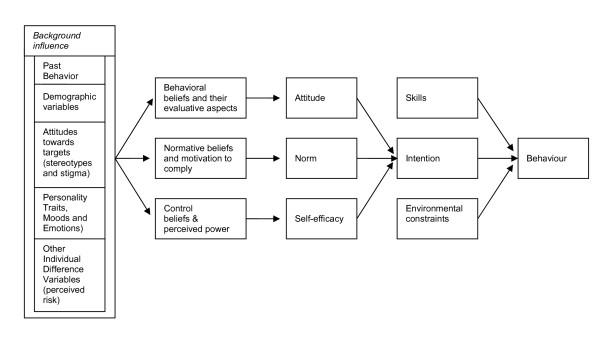
**Integrated Model **[50].

In an effort to integrate the leading theories on behavioral prediction and behavioral change, Fishbein developed the Integrative Model of Behavioral Prediction [30,31] (Figure 1). This model functioned as the theoretical framework for our study of the determinants of STI testing in vocational school students. The Integrative Model synthesizes aspects of the Theory of Reasoned Action (TRA), the Theory of Planned Behavior (TPB) [32], Social Cognitive Theory (SCT) [33] and the Health Belief Model (HBM) [34]. The model first suggests that behavior is caused by intention when skills to cope with environmental constraints are sufficient. The most proximal determinants of intention are attitude, perceived norms and self efficacy. The proximal determinants themselves are functions of sets of underlying beliefs. Background variables are considered to have an indirect influence on the proximal determinants through their impact on beliefs. Possible background variables for participating in STI testing among adolescents include risk perception (e.g. severity and worry) [26,35], mood and emotions (e.g. shame), stigma attached to testing [27,36], knowledge, interventions that promote testing, perceived accessibility and availability of testing services (e.g. the availability of oral and rapid test methods as well as free testing services) [26,37], demographic variables and past testing behavior. In predicting intentions, past behaviour can also have a direct influence on intentions and has some variance in common that is not shared by attitude, perceived norms and perceptions of control [38]. The purpose of this study was to assess sexual risk behavior and STI testing, and to identify psychosocial and environmental factors predicting STI testing intention among sexually experienced adolescents attending vocational schools. The Integrative Model functioned as our theoretical framework.

## Methods

### Participants

Between October and December 2006, students attending one of five vocational schools located in the Rotterdam area were invited to participate in this cross-sectional study. These schools belong to two large school systems that were approached by the municipal health service. After consultation with the school administrators, the various schools in those two systems were approached. A convenience sample of 5 of the 39 available schools was selected such that the different types and levels of education were represented. All first and second year students attending classes on the days the survey was conducted were asked to participate. Class size varied from 3 to 35 students, with an average of 15 students. Of the 972 students approached for participation, 918 (94.4%) agreed to participate and, of those, 778 (84.7%) completed the survey. About a third of the attrition between consent and survey completion was attributable to technical problems with the host server (n = 46). Also, participants younger than 15 (n = 2) or older than 25 (n = 20) were excluded after survey completion, thus resulting in a usable sample of 756 students. Because our study pertained to sexual behavior, only those students that had already had vaginal or anal intercourse were included in the analyses (66.3%; n = 501).

The students that agreed to participate but did not (fully) complete the survey were similar to the 756 for which complete data was available in terms of their ethnicity and religion but not in terms of sex, age and educational attainment. The drop-outs were more frequently male (74.4% versus 47.4%; χ^2 ^(1, *N *= 889) = 33.21, *p *< .001), older (18.8 versus 18.0 years; *t*(887) = 3.68, *p *< .001), better educated (57.6% highest levels versus 47.5%; χ^2 ^(1, N = 889) = 4.20, *p *< .05) and studying Information and Communication Technology (ICT) (49.6 versus 12.8%; χ^2 ^(5, *N *= 856) = 107.95, *p *< 0.001). This is, at least in part, because substantial technical problems occurred on the day that ICT classes participated.

The gender distribution in the 756 students was comparable to that of the general student population at vocational schools in Rotterdam. However, students of non-Dutch ethnicity were somewhat underrepresented. In our study, 62% of participants had a non-Dutch ethnicity while this percentage is 68% in the general student population [39]. Students without sexual experience were also more frequently female (69.8% versus 43.9%; χ^2 ^(1, *N *= 756) = 45.44, *p *< 0.001), younger (17.4 versus 18.3 years; *t*(754) = -6.26, *p *< 0.001), Muslim (45.1% versus 17.4%; χ^2 ^(3, *N *= 756) = 67.72, *p *< 0.001) and of Turkish or Moroccan descent (23.5% versus 10.4% and 16.5% versus 5.5%, respectively; χ^2^(6, *N *= 756) = 68.5, *p *< 0.001).

Socio-demographic characteristics of the final sample are presented in Table 1. The mean age was 18.3 (range 16-25) and 56% were male. The majority were of non-Dutch ethnicity (62%), namely Surinamese (16%), Antillean (12%), Turkish (10%), Cape Verdean (6%) and Moroccan (5%).

**Table 1:** Characteristics of vocational school students with sexual experience according to gender

	N = 501	%	Boys N = 281	%	Girls N = 220	%
Age - mean (SD)range	18.3 (2.1) 16-25		18.1 (1.7)		18.6 (2.4)	
Religion						
None	208	41.5	116	41.3	92	41.8
Christian	175	34.9	82	29.2	93	42.3
Muslim	87	17.4	64	22.8	23	10.5
Hindu	26	5.2	16	5.7	10	4.5
Other	5	1.0	3	1.1	2	0.9
Level of education^1^						
1+2	265	52.9	144	51.0	121	55.0
3+4	236	47.1	137	49.0	99	45.0
Program						
Health	105	21.0	15	5.3	90	40.9
Technical studies	103	20.6	100	35.6	3	1.4
Economics	92	18.4	35	12.5	57	25.9
Sports	79	15.8	61	21.7	18	8.2
ICT ^2^	68	13.6	65	23.1	3	1.4
Welfare	54	10.8	5	1.8	49	22.3
Ethnic background ^3^						
Dutch	190	37.9	109	38.8	81	36.8
Surinamese	80	16.0	41	14.6	39	17.7
Antillean	61	12.2	25	8.9	36	16.4
Turkish	52	10.4	40	14.2	12	5.5
Cape Verdean	28	5.6	7	2.5	21	9.5
Moroccan	23	4.6	19	6.8	4	1.8
Other 'Western'	28	5.6	15	5.3	13	5.9
Other 'non-Western'	39	7.8	25	8.9	14	6.4

^1 ^The Dutch vocational school system has 4 levels of education, 1 is the lowest while 4 is the highest. Level 2 or more is required to qualify for a skilled job

^2^ICT = Information and Communication Technology

^3^Based on the definition of Statistics Netherlands: a person whose parents were both born in the Netherlands is classified as ethnic Dutch, a person of whom at least one parent was born abroad is classified with a non-Dutch ethnic background.

### Procedure

Recruitment occurred in vocational school classrooms. The study was first introduced by a teacher and then further described by a member of the research team. The students were asked to provide consent on the first page of the interactive online questionnaire before completing the Dutch language survey which comprised 162 items (Additional file 1). Administration of the survey took between 25 and 50 minutes. To ensure confidentiality and avoid discipline problems (e.g. discussing the answers and looking at each others screens), the teacher and a research team member supervised the procedure. As a reward, the students were offered a pen and a condom. Approval for this study was provided by the ethics committee at Maastricht University's Faculty of Psychology and Neuroscience.

Prior to initiation of the study, we contacted experts in behavior change, adolescence and STI prevention and asked them to assess the validity of the survey. Teachers were also invited to review the instrument and make recommendations for its modification. In order to adequately tailor the survey items to the linguistic capacities and cultural background of the participants, we pilot-tested the survey with 13 vocational students of various ethnicities. The instrument's reliability was subsequently examined by performing a test-retest procedure (one week interval) with 39 students. The test-retest correlations were above 0.5 on all items except for one item measuring attitude, two items measuring stigma, two items measuring perceived norms and two items measuring knowledge. The questionnaire was adapted on the basis of these results. More specifically, because students reported that too much repetition was present in the survey and that some questions were too difficult and abstract, we removed one hypothetical situation and two items (measuring direct attitude and determinants of condom use for anal sex), reformulated some questions so that they would be shorter and easier to read and added more detailed explanations where needed.

### Measures

#### Demographic variables

Socio-demographic variables measured included gender, age, religion, type and level of vocational course and ethnic background.

#### Sexual behavior, condom use and STI testing

Items measuring: 1) relationships; 2) sexual experience with oral, vaginal and anal sex; 3) condom use; 4) number of steady and casual sexual partners in the previous 12 months; 5) lifetime steady and causal sexual partners; and 6) experience with STI testing were derived from a Dutch national study on sexual behavior among adolescents [5]. Students who were sexually active in the past 12 months were also asked to respond to items on condom use over the past 12 months. The recall period of 12 months was chosen as 12 months is considered sufficient to reflect the actual lives and relationships of adolescents [40]. Condom use during vaginal and anal sex in both steady and casual relationships was measured using a five-point scale ranging from 'never' to 'always'.

#### Psychosocial constructs

All items assessing psychosocial constructs were measured on bipolar five-point scales. When measured with multiple items, the mean score for each construct was calculated but only after internal consistency was established (Table 2).

**Table 2:** Scales and items used to measure (psychosocial) correlates of STI testing (n = 501)

Determinant (range)	No. of items	Internal consistency (a)	Total mean Score N = 501	SD	Boys Mean score N = 281	SD	Girls Mean score N = 220	SD
Intention to test *	2	0.75	0.10	1.01	0.01	1.03	0.21	0.99
Attitude toward testing **	4	0.82	0.44	0.92	0.31	0.90	0.60	0.92
Advantages of testing ***	10	0.83	0.79	0.67	0.68	0.76	0.92	0.50
Disadvantages of testing ***	7	0.71	-0.19	0.70	0.08	0.75	0.32	0.62
Injunctive norm - friends and parents **	4	0.71	0.09	0.81	0.00	0.80	0.21	0.81
Support - friends and parents **	4	0.73	0.71	0.79	0.63	0.84	0.81	0.71
Test behavior - partner ***	1		-0.26	1.02	-0.31	0.89	-0.21	1.18
Injunctive norm - partner *	1		-0.14	0.90	-0.06	0.76	-0.23	1.04
Support - partner	2	0.69	0.62	0.86	0.43	0.77	0.87	0.92
Descriptive norm - friends	1		-0.34	1.03	-0.40	1.04	-0.27	1.00
Self efficacy ***	8	0.86	0.08	0.79	0.22	0.79	-0.11	0.74
Perceived susceptibility ***	5	0.88	0.88	0.82	0.67	0.87	1.15	0.67
Perceived severity	2	0.89	1.79	0.57	1.75	0.65	1.83	0.45
Perceived shame ***	5	0.86	0.45	0.95	0.23	0.96	0.73	0.86
Perceived stigma	6	0.84	-0.21	0.86	-0.22	0.86	-0.20	0.86
Knowledge ***	10	0.76	7.23	2.50	6.72	2.66	7.87	2.1
Test site characteristics **	8	0.80	0.43	0.70	0.34	0.73	0.54	0.64

Note: Differences in scores between boys and girls were tested using independent sample t-tests by gender

* p < .05 (for gender difference); ** p < .01 (for gender difference); *** p < .001 (for gender difference); all items were measured on scales ranging from -2 to +2 (fully disagree/certainly not to fully agree/certainly) except for knowledge that was measured with a range of 0 to 10 (numbers represent number of right answers).

#### Integrative Model, proximal determinants

##### Behavioral intention

Behavioral intention, direct attitude toward testing and perceived norm scales were developed in accordance with TPB [38] and used hypothetic situations (i.e. intention to test "after intercourse with someone without a condom" and "before having sex without a condom in a new relationship"). These hypothetical situations are in line with the prevention messages currently provided to young heterosexual people in the Netherlands. Behavioral intention was assessed by two items that posed hypothetical situations and then asked if the participant would go for an STI test in that situation.

##### Attitude

Direct attitude toward testing was measured by four items that asked participants how pleasant or unpleasant, wise or unwise they found STI testing to be in each of the hypothetic situations. Indirect attitude toward testing was indexed by means of 17 behavioral beliefs about the advantages and disadvantages of testing (e.g. disadvantage: "Getting an STI test means that I don't trust my partner enough"; advantage: "Getting an STI test is taking responsibility for my own health"). Thirteen items were adapted from a survey that measured HIV testing behavior among MSM [41] (N = 3500; advantages: α = .63; disadvantages: α = .72). Four items were constructed by the authors and informed by data obtained from the previously mentioned semi-structured interviews with the target population.

##### Perceived norms and social support

Perceived norms toward testing were assessed by four items that measured expected social approval from friends and parents in hypothetic situations (e.g. "You have a new boyfriend/girl friend and you want to have sex with her/him without a condom. Do your friends/parents think that you should first take an STI test?"; "Do your friends/parents think that you should take an STI test before you have unprotected sex?"). Social support from friends and parents was also assessed by four items adapted from previous research on HIV testing among Dutch MSM [42] (e.g. "If I take an STI test/If I receive unfavorable test results, my friends/parents will support me"). Testing behavior and perceived norm of partner were each assessed by one item ('Has your partner been tested for an STI?' and 'Does your partner think that you should get an STI test?'), while social support from partner was measured by two items (e.g. "Do you think your partner will support you if you take an STI test/after an unfavorable test result?"). Descriptive norm was measured by one item that asked participants to estimate how many of their friends have taken an STI test ('none of my friends' to 'all of my friends'). A total of eight self efficacy items asked participants how difficult ('very difficult' to 'very easy') they found performing a number of tasks involved in getting an STI test to be (e.g. discussing sexual behavior with a nurse or physician; waiting for test results). These self efficacy measures were also adapted from a study on HIV testing behavior among Dutch MSM [41] (N = 3500, α > .75).

#### Background variables

##### Susceptibility

Perceived susceptibility was measured by five items that asked participants to estimate their risk ('not likely at all' to 'very likely') of getting an STI from unprotected intercourse with five types of partners. Two of the five items were previously used by Fulpen [43] (i.e. "Imagine you have unprotected sex (sex without a condom) with someone you do not know very well/you have been a relationship with for a couple of months. How likely are you to get an STI?"). We added three other partner types, namely someone you met through friends, someone you met on holiday and someone you met on the Internet. Perceived severity was measured by asking participants to report their agreement ('totally agree' to 'totally disagree' with two items, namely "I think it would be really terrible to have an STI" and "I think it would be really terrible to have HIV". These items were derived from a Dutch national study on sexual behavior among adolescents [5] (N = 9250, α = .78).

*Shame *was measured by five items reflecting participants' sense of shame and related negative affect states (e.g. "If you had an STI, would people avoid you?") while six perceived stigma items reflected participants' expectations of negative interactions and judgment associated with STI (e.g. "If you had an STI, would you feel ashamed?"). Both the shame and stigma scales were previously employed by Cunningham in research conducted with African American adolescents [27] (N = 142, shame scale: α = .90; stigma scale: α = .89).

##### Knowledge

STI knowledge was measured by ten true/false/I don't know items (e.g. "You can prevent an STI by washing well after sex"; "Anal sex without a condom increases your risk for getting an STI"). These items were derived from two Dutch national studies [5,44]. We also added three new items because the interviews that informed the survey suggested that these items are relevant. Two of the three new items pertained to risks associated with anal and oral sex and the last item measured the perceived relationship between looks and STI status.

##### Test site characteristics

Seven items measured the degree to which students' considered various aspects of testing services (e.g. you can see a doctor who does not know you, results are provided relatively quickly) to be important (5 point scale from 'not important at all' to 'very important'). These items were generated on the basis of the previously mentioned interview data.

### Data analyses

Analyses were conducted with SPSS version 15.0. Linear regression analyses were employed to identify correlates of intention to get an STI test. In univariate regression analyses, associations between intention, on the one hand, and age, ethnicity, religion and sex, on the other, were assessed and significant predictors were entered into the regression model. Then associations between intention and two behavioral factors, namely testing history and not using a condom in a steady relationship, were assessed. Testing history was found to be significant and added to the model. We subsequently employed the backward procedure to test the psychosocial variables and test site characteristics. Interactions between gender and the psychosocial determinants were also explored. Additionally, we calculated Pearson's correlations for direct attitude toward testing and behavioral beliefs and for intention and behavioral beliefs.

## Results

### Sexual behavior and condom use

Behavioral characteristics of the sample are reported in Table 3. The proportion of students reporting having engaged in anal intercourse differed significantly between boys (28.5%) and girls (17.7%; χ^2^(1, *N *= 501) = 7.86, *p *< .01) while girls reported significantly more experience with oral sex (78.2%) than boys (59.4%; χ^2 ^(1, *N *= 501) = 19.83, *p *< .001). In total, 42% of the students (*N *= 321) reported having had more than one sexual partner in their lifetime. The number of lifetime partners for vaginal intercourse differed significantly between boys and girls (boys 7.1 versus girls 3.3; *t*(441) = 5.5, *p *< .001). The proportion of boys reporting consistent condom use during vaginal sex with a steady partner was 41.1% compared to 24.2% for girls, χ^2 ^(1, *N *= 368) = 11.89, *p *< .001. For vaginal sex with casual partners, 57.9% reported using condoms consistently. No significant difference between boys and girls was found. Among participants that had had anal sex, the majority reported inconsistent condom use. This was the case for both steady and casual partners. Students with a non-Dutch ethnicity reported more risk behavior than Dutch students. They reported a mean of 6.4 lifetime partners for vaginal intercourse versus the mean score of 3.9 among Dutch students; *t*(441) = 2.7, *p *< .01). They also reported less consistent condom use with steady partners (27.9% versus 40.3%, χ^2 ^(1, *N *= 368) = 6.19, *p *< .05).

**Table 3:** Behavioral characteristics of vocational school students with sexual experience according to gender

	N = 501	%	Boys N = 281	%	Girls N = 220	%
Involved in steady relationship ***	265	52.9	119	42.3	146	66.4
						
Sexual orientation						
Heterosexual	491	98.0	275	97.9	216	98.2
Homosexual	4	< 1	1	< 1	3	1.4
Bisexual	3	< 1	2	< 1	1	< 1
Don't know yet	3	< 1	3	1.1	0	0
						
Sexual behaviors						
Vaginal sex	496	99.0	279	99.3	217	98.6
With steady partner(s) **	368	73.5	190	67.6	178	80.9
With casual partner(s) ***	247	49.3	178	63.3	69	31.4
Mean no. of lifetime partners vaginal intercourse (SD) *** (n = 443, n_male _= 235, n_female _= 208)^1^	5.3 (7.8)		7.1 (9.4)		3.3 (4.7)	
						
Anal sex **	119	23.8	80	28.5	39	17.7
With steady partner(s) *	75	15.0	52	18.5	23	10.5
With casual partner(s) ***	50	10.0	42	14.9	8	3.6
Mean no. of lifetime partners anal intercourse (SD) * (n = 99 n_male _= 64, n_female _= 35)^1^	2.1 (2.9)		2.4 (3.5)		1.5 (1.3)	
						
Oral sex ***	339	67.7	167	59.4	172	78.2
Mean no. of partners oral sex in past 12 Months (SD) *** (n = 337, n_male_= 165, n_female _= 172)^1^	2.3 (4.4)		3.3 (5.9)		1.3 (1.6)	
						
*Testing behavior*						
Ever tested for HIV ***	48	9.6	13	4.6	35	15.9
Positive diagnosis for HIV	0	0.0	0	0.0	0	0.0
Ever tested for other STI **	77	15.4	32	11.4	45	20.5
Positive diagnosis for STI	18	23.4	4	12.5	14^2^	31.8
						
*Condom use in past 12 months*						
Always used a condom during vaginal sex with steady partner^3 ^***	121	32.9	78	41.1	43	24.2
Always used a condom during vaginal sex with casual partner^4^	143	57.9	105	59.0	38	55.1
Always used a condom during anal sex with steady partner^5^	22	29.3	17	32.7	5	21.7
Always used a condom during anal sex with casual partner^6^	20	40.0	17	40.5	3	37.5

* p < .05 (for gender difference); ** p < .01 (for gender difference); *** p <.001 (for gender difference)

^1 ^Total does not add up to the total of participants reporting the given sexual experience because of missing values and case exclusion due to inconsistencies in reported number of partners (e.g. smaller number of lifetime partners than the number of partners in the past year).

^2 ^One female respondent did not report her test result.

^3 ^Percentage represents condom use during vaginal sex with a steady partner/number of participants that have had vaginal sex with a steady partner.

^4 ^Percentage represents condom use during vaginal sex with a casual partner/number of participants that have had vaginal sex with a casual partner.

^5 ^Percentage represents condom use during anal sex with a steady partner/number of participants that have had anal sex with a steady partner.

^6 ^Percentage represents condom use during anal sex with a casual partner/number of participants that have had anal sex with a casual partner.

### Past STI testing

Among students with sexual experience (vaginal or anal), girls were significantly more likely to have gone for an HIV or other STI test at some point in time prior to their participation in this study (HIV: 4.8% of boys versus 16.0% of girls, χ^2 ^= 17.38, *p *< .001; other STI: 11.4% of boys versus 20.5% of girls, χ^2 ^= 7.80, *p *< .01). About one in every four students that had previously had an STI test done reported having tested positive for an STI with girls being more likely to have tested positive than boys (31.8% versus 12.5%, χ^2^= 3,8; *p *= 0.06). With respect to testing history, no differences in testing prevalence and positive diagnoses were found between students with a non-Dutch ethnicity and Dutch students. Students with a non-Dutch ethnicity utilized free testing services offered by the municipal health services more often while Dutch students were more likely to have the test done through their general practitioner.

### Psychosocial determinants of STI testing

Table 2 displays, among other things, the mean scores for the psychosocial determinants of STI testing. Overall, the sample's scores on psychosocial determinants corresponded with scale means except with respect to perceived severity (sample scored substantially more positively) and with respect to perceived susceptibility, social support and the advantages of testing (sample scored relatively more positively). Also, girls scored more positively than boys on all psychosocial determinants except self efficacy.

The correlation between the sum score for behavioral beliefs and the sum score for direct attitude, which measured how pleasant or wise students considered STI testing to be, was .23. In terms of individual beliefs, we found that 11 were significantly correlated with intention. These positive beliefs pertained to the advantages of knowing one's STI status, the benefits associated with being able to receive timely treatment, taking responsibility, and showing your partner that you are serious about the relationship. Negative beliefs were the fear of receiving a positive test result.

#### Intention to participate in STI testing

Table 4 reports the results of the multiple regression analyses predicting intention to participate in STI testing, while Table 5 presents correlations between the variables. The final model explained 39% of the total variance associated with intention to test. When corrected for age, sex, ethnicity and testing history, attitude was found to be the strongest predictor. Perceived norms (friends and family's injunctive norms and friends' descriptive norms) and perceived susceptibility were also significant predictors of intention to test, as was accessibility of testing facilities. Interactions between psychosocial correlates and gender were non-significant.

**Table 4:** Pearson's correlations (r) with intention and standardized regression coefficients (β) for predictors of intention (N = 501).

		*r*	β	p
Constant				0.00
Age		0.21 **	0.09	0.02
Sex (female)		0.10 *	-0.07	0.05
Etnicity^a:: ^Surinamese		n.s.	-0.00	0.96
Etnicity^a: ^Turkish		n.s.	0.03	0.57
Etnicity^a: ^: Moroccan		n.s.	0.02	0.68
Etnicity^a: ^: Antillean		0.13 ***	0.06	0.14
Etnicity^a: ^: Cape Verdean		0.11 *	0.03	0.51
Etnicity^a: ^: other non-Western		n.s.	0.05	0.19
Religion^b^: Christian		0.13 **	0.01	0.76
Religion^b^: Muslim		n.s.	0.04	0.55
Religion^b: ^Other		n.s.	0.04	0.40
Testing history previous STI test		0.25 **	0.13	0.00
Attitude		0.49 **	0.35	0.00
Injunctive norm - friends and parents		0.38 **	0.18	0.00
Descriptive norm - friends		0.26 **	0.10	0.01
Perceived susceptibility		0.33 **	0.15	0.00
Test site characteristics		0.22 **	0.11	0.00
				
	R^2^	0.39		

* p < .05; ** p < .01; *** p < .001.

^a ^Reference category: Dutch; ^b ^Reference category: no religion.

**Table 5:** Correlations between (psychosocial) correlates of STI testing

		1	2	3	4	5	6	7	8	9	10	11	12	13	14	15	16	17
1	Intention	1.00																
2	Attitude	.49**	1.00															
3	Advantages	.12*	.22**	1.00														
4	Disadvantages	-.02	.02	.53**	1.00													
5	Injunctive norm friends and parents	.38**	.30**	.13**	.05	1.00												
6	Supp friends and parents	.02	.11*	.16**	-.09	.26**	1.00											
7	Test behavior partner	.17 **	.14**	.03	-.08	.22**	.01	1.00										
8	Injunctive norm partner	.11*	.06	.01	.00	.21**	-.03	.27**	1.00									
9	Support partner	.05	.15**	.20**	.08	.08	.27**	-.18**	-.12*	1.00								
10	Descriptive norm friends	.26**	.10*	-.07	-.08	.39**	-.02	.23**	.13**	.00	1.00							
11	Self efficacy	.11*	.09*	-.11*	-.45**	.12*	.19**	.07	.01	.00	.14**	1.00						
12	Susceptibility	.33**	.32**	.19**	.08	.18**	.11*	.06	.05	.17**	.07	-.03	1.00					
13	Severity	.06	.04	.17**	.06	.12*	.21**	-.01	.00	.09*	.02	.00	.19**	1.00				
14	Shame	.19**	.18**	.15**	.26**	.18**	.11*	.00	.04	.12*	.08	-.19**	.31**	.22**	1.00			
15	Stigma	.08	.04	-.03	.18**	.05	-.20**	.03	.06	-.05	.15**	-.18**	.16**	.11*	.42**	1.00		
16	Knowledge	.03	.13**	.29	.05	.08	.31**	-.02	-.04	.23**	-.13**	.03	.03**	.21**	.07	-.08	1.00	
17	Test site characteristics	.22**	.19**	.11*	.14**	.14**	.07	-.01	.06	.03	.04	-.06	.22**	.11*	.32**	.17**	.06	1.00

## Discussion

This study has demonstrated a high prevalence of unsafe sex and low STI testing rates among sexually active students at vocational schools in the Netherlands. We examined if, and to what degree, the variables posited by the Fishbein's Integrative Model could predict students' intentions to participate in STI testing. We found that the Integrative Model variables successfully explained 39% of the variance and that intention to test was very low.

Similar to other studies [11,36,41,45], we found that the proximal determinants intention, attitude and norms were significant predictors of STI testing. In contrast with other research that focused on HIV testing [11,26,45,46], we found that self efficacy was not a significant predictor. This is, however, consistent with other research on HIV testing conducted in the Netherlands [41]. It is possible that this finding is attributable to the fact that participating in STI testing in the Netherlands is relatively easy to do. Test sites are readily available to adolescents.

In accordance with other studies, we found perceived susceptibility but not perceived severity to be a predictor of intention to participate in an STI test [25,47]. Most of the students thought that STI were very severe (mean score of 1.78 at a scale of -2/+2; SD = 0.57). As a consequence, the data on severity were negatively skewed (scores clustering at the higher end of the scale). This situation statistically hinders the determination of a relationship between severity and intention. Perceived susceptibility was also found to be very low, given the self reported risk behavior. According to national guidelines, the sexual behavior of two thirds of the boys and half of the girls warrants an STI test [48] because students either had unprotected sexual intercourse with a casual partner in the past year (17% of the boys; 9% of the girls) or two or more sexual partners in their lifetime (66% of boys; 51% of girls) or both. Despite the significant risk, only 15% had previously been tested for an STI.

Also, our finding that easy access to testing facilities is a significant predictor of intention to test is consistent with studies on HIV testing in young adults [26,37]. Providing testing for free, ensuring anonymity and confidentiality, offering testing services outside of office hours and providing options with regard to how one can receive one's test results all appear to contribute to intention to get an STI test.

In this study, girls reported having previously tested for an STI more often than boys. Their intention to test was also higher. That girls have more experience with STI testing may be due to the fact that girls tend to contact health care providers for reproductive health matters more than boys. They often visit their GP for a contraceptive prescription and thus have more opportunities to request an STI test than boys. Girls also scored higher than boys on all behavioral determinants of testing except self efficacy. However, despite of these differences in scores, there were no gender differences regarding which determinants were predictive for the intention to test. This is in contrast to other studies that suggest that behavioral determinants for testing are different for boys and girls. Such studies have indicated that fear of stigma might hinder girls but not boys in seeking STI-related care [23,27].

The majority of the sample were of non-Dutch ethnicity (first and second generation migrants), which is typical for vocational students in Rotterdam and in other urban centers in the Netherlands. Our study did not demonstrate significant difference between students of non-Dutch ethnicity and Dutch students regarding testing behavior and the determinants of STI testing. This suggests that an intervention promoting testing that is geared to vocational students does not necessarily need to be tailored to differences in ethnicity. Such an intervention should, however, be tailored to risk behavior and the determinants of STI testing investigated in this study.

In short, we conclude that intention to participate in STI testing among this population of adolescents is primarily driven by attitude but also by norms. Because most students were unaware of the relevance of STI testing, we recommend that interventions first target testing awareness and then seek to establish a positive attitude toward testing via the specific beliefs that underlie attitudes toward testing [49]. For example, interventions may benefit from focusing on positive beliefs such as the advantages of a timely treatment, taking responsibility for one's health and communicating a positive message to one's partner. In order to increase the accessibility of pre-existing positive beliefs, priming strategies could be employed. Priming strategies attempt to increase the accessibility of specific beliefs in order to increase their influence on intentions, even in the absence of belief change [50]. An example of a priming strategy would be to incorporate certain beliefs in a movie and then discuss these with the target group afterwards. Additionally, the provision of information on the treatment and counseling procedure may function to remove worry about negative test results. Further, in order to establish positive social norms toward STI testing, the role of friends and parents should be addressed. A possible intervention could entail encouraging students to mobilize social support from friends. Another appropriate method is modeling which can change not only perceptions of social influence but also the social environment by changing the norms of peers [7]. Perceived susceptibility should be also addressed. Adequate perceptions of risk can be promoted through the personalization of risk. Lastly, easy access to testing facilities should be promoted and adolescents should be made aware of the availability of testing services.

The validity of this study is strengthened by its high response rate and the fact that the survey was systematically developed and based on both theory and evidence. A limitation to be considered is the size of the sample employed for the test-retest procedure (*N *= 39). Another limitation is the cross-sectional design of this study. Because of this, causality cannot be determined. However, in light of existing theory, the proposed causal direction is relatively probable. Furthermore, the correlation found between direct attitude toward testing and the sum score for beliefs was low (*r *= .23) thus indicating that we were unable to identify the most salient beliefs underlying students' attitude toward STI testing [49]. This is perhaps because we did not measure outcome evaluations. According to theory, beliefs should be multiplied by their outcome evaluations [38]. Outcome evaluations assess the item's evaluative implication for the individual and can be particularly useful for items that are ambiguous. For example, the outcome evaluation accompanying the item "getting an STI test is taking responsibility for my own health" would assign the degree to which the participant thinks that taking responsibility for one's own health is important. Another possibility is that we failed to measure all relevant beliefs or, because of a lack of compatibility, we failed to measure them correctly [49]. The measure of direct attitude toward testing used hypothetical situations while the measure of individual beliefs did not. However, scores on intention and its behavioral determinants were relatively low thus suggesting that students had not (yet) formed strong opinions regarding testing and that we measured non salient or less readily available beliefs. Despite these limitations, our findings reveal information on determinants of STI testing for an important target group. They thereby contributed to evidence that is essential for developing prevention interventions.

## Conclusions

The present study provides important and relevant empirical input for the development of health promotion interventions that promote STI testing among adolescents attending vocational schools in the Netherlands. Theory and evidence on how health behavior can best be changed can contribute to the development of an effective intervention that promotes STI testing. According to the IM protocol [7], effective strategies and methods can be chosen for every important and changeable determinant when designing the intervention. The results of this study suggest that interventions promoting STI testing should aim to change attitudes, address social norms and elevate personal risk perception for STI. Additionally, the accessible nature of testing facilities should be promoted.

## Competing interests

The authors declare that they have no competing interests.

## Authors' contributions

MW conceived of the study, participated in its design, carried out the study, performed the analyses and drafted the manuscript. GK discussed interpretation of results and provided comments on the manuscript. JM helped conceive the study, participated in the design of the study and provided comments on the manuscript. OZ participated in the design of the study, helped draft the manuscript and coordinated the study. All authors read and approved of the final manuscript.

## Pre-publication history

The pre-publication history for this paper can be accessed here:

http://www.biomedcentral.com/1471-2458/10/725/prepub

## Supplementary Material

Additional file 1 **Questionnaire**. Click here for file
