# Supplementary material for: Correlates of STI testing among vocational school students in the Netherlands
Source: BMC Public Health. 2010 Nov 24;10:725. doi: 10.1186/1471-2458-10-725 (PMC3009645; doi:10.1186/1471-2458-10-725)
Supplement: Additional file 1 — Questionnaire. [file 1471-2458-10-725-S1.DOC]

# Part 1: Personal situation.

1. **What is your age?** …………… years

1. **Are you a man or a women?**

1 □ man

2 □ vrouw

1. **In which country were you born?**

1 □ The Netherlands

2 □ Turkey

3 □ Morocco

4 □ Surinam

5 □ The Dutch Antilles or Aruba

6 □ Cape Verdean Islands

7 □ another country:………………………….

1. [If not 3=1**] How old were you when you migrated to the Netherlands?**

…………… years

1. **In which country was your mother born?**

1 □ The Netherlands

2 □ Turkey

3 □ Morocco

4 □ Surinam

5 □ The Dutch Antilles or Aruba

6 □ Cape Verdean Islands

7 □ another country:………………………….

1. **In which country was your father born?**

1 □ The Netherlands

2 □ Turkey

3 □ Morocco

4 □ Surinam

5 □ The Dutch Antilles or Aruba

6 □ Cape Verdean Islands

7 □ another country:………………………….

1. **What is your postal code? Only fill in the numbers!**

|  |  |  |  |
| --- | --- | --- | --- |

1. **Are you religious?**

1 □ yes

2 □ no **continue with question 10**

1. **Wat is your religion?**

1 □ Roman Catholic

2 □ Protestant

3 □ Dutch reformed

4 □ Muslim

5 □ Hindu

6 □ Jewisch

7 □ Buddhism

8 □ other:.............................................

1. **In which year are you?**

1 □ first

2 □ second

3 □ third

4 □ fourth

1. **What educational program do you attend?**

| 1 | Administration |
| --- | --- |
| 2 | Information and Communication Technology |
| 3 | Security |
| 4 | Bookkeeping |
| 5 | Construction |
| 6 | Bakery |
| 7 | Communication |
| 8 | Service industries |
| 9 | Healthcare |
| 10 | Sales |
| 11 | Tourism, hotel and catering |
| 12 | Fashion |
| 13 | Painting |
| 14 | Sports |
| 15 | Technique |
| 16 | Extermal care |
| 17 | Welfare |
| 18 | Other: ........................... |

# Part 2: Relationships

1. **Did you ever have a steady relationship (steady boyfriend-girlfriend)?**

1 □ no

2 □ yes, once

3 □ yes, more than once

1. **At this moment, do you have a steady relationship?**

1 □ yes

2 □ no

1. **Do you have a steady relationship with a boy or a girl?**

1 □ a boy

2 □ a girl

1. **What is the duration of this relationship?**

**……. Weeks**

**or ……. Months**

**or ……. years (and ..........months)**

# Part 3 Sexual Transmittable Infections (STI)

What are STI?

STI: Sexual transmittable infections (venereal diseases). Diseases that you may get if you have unsafe sex. Examples are Chlamydia, Gonorrhea, Syphilis, Herpes or HIV.

HIV: the virus that causes Aids.

1. **Do you think the following statements are right or wrong? When you don’t know the answer, please do not guess the answer, but choose “I don’t know” This is not an exam!**

|  |  | **right** | **wrong** | **I don’t know** |
| --- | --- | --- | --- | --- |
| **1** | When taking the contraceptive pill it is less likely that you catch an STI | □ | □ | □ |
| **2** | You can prevent an STI by washing well after sex | □ | □ | □ |
| **3** | Even if you have no physical complaints, you can have an STI | □ | □ | □ |
| **4** | Certain STI can cause infertility in women | □ | □ | □ |
| **5** | Most STI will just go away automatically | □ | □ | □ |
| **6** | By having had unsafe sex only once, you will not get an STI | □ | □ | □ |
| **7** | By having oral sex without a condom you can get an STI | □ | □ | □ |
| **8** | By having anal sex without a condom you can get an STI | □ | □ | □ |
| **9** | You can see from someone’s appearances if he or she is infected with HIV | □ | □ | □ |
| **10** | Some STI you will never get rid of. | □ | □ | □ |

1. **Do you agree with the following statements­?**

|  | | **completely agree** | **agree** | **not agree/not disagree** | **disagree** | **completely disagree** |
| --- | --- | --- | --- | --- | --- | --- |
| 1 | I think it would be really terrible to have HIV | □ | □ | □ | □ | □ |
| 2 | I think it would be really terrible to have another STI | □ | □ | □ | □ | □ |

1. **Do you know someone who has had an STI ?**

□ yes

□ no

**Contraceptive**: something you use to prevent pregnancy. For example: condom, contraceptive pills, the spiral, contraceptive plasters

1. **[for girls] Do you use contraceptives? You can choose more options**

□ no

□ yes, contraceptive pills

□ yes, condoms

□ yes, injectable hormonal contraceptives

□ yes, contraceptive plasters

□ yes, vaginal ring (NuvaRing)

□ yes, hormonal contraceptive implant (Implanon )

□ yes, the spiral

□ yes, something else

1. **[for boys] Does your steady partner use contraceptives?**

□ no

□ yes, contraceptive pills

□ yes, condoms

□ yes, injectable hormonal contraceptives

□ yes, contraceptive plasters

□ yes, vaginal ring (NuvaRing)

□ yes, hormonal contraceptive implant (Implanon )

□ yes, the spiral

□ yes, something else

□ I don’t know

# Part 4: Sexual experiences

1. **Do you feel sexual attracted to men, women, or both?**

1 □ only to men

2 □ predominantly to men, but also to women

3 □ to men as much as to women

4 □ predominantly to women, but also to men

5 □ only to women

6 □ I don’t know yet

1. **Have you ever had sexual intercourse?**

1 □ no **Continue with question 31**

2 □ yes

1. **So far, with how many different persons did you have sexual intercourse?**

with ……. different persons

Steady partner: someone you have a steady relationship with (go steady with) and have sex with. If you are married, your spouse is your steady partner.

Casual partner: someone you have sex with (once, or several times), but you with whom you have no steady relationship.

1. **Did you have vaginal intercourse with a steady partner in the past 12 months?**

□ yes

□ no **Continue with question 27**

1. **With how many steady partners did you have vaginal intercourse in the past 12 months?**

with ……. steady partners

1. **How often did you use condoms during vaginal intercourse with a steady partner in the past 12 months?**

□ never

□ most of the times not

□ sometimes yes, sometimes not

□ most of the times

□ always

1. **Did you have vaginal intercourse with a casual partner in the past 12 months?**

□ yes

□ no **Continue with question 30**

1. **With how many casual partners did you have vaginal intercourse in the past 12 months?**

with ……. casual partners

1. **How often did you use condoms during vaginal intercourse with a casual partner in the past 12 months?**

□ never

□ most of the times not

□ sometimes yes, sometimes not

□ most of the times

□ always

1. **The person you had sex with the last time, what was his or her ethnic background?** 1 □ Dutch

2 □ Turkisch

3 □ Maroccon

4 □ Surinamese

5 □ Dutch Antillian or Aruban

6 □ Cape Verdian

7 □ other:………………………….

(ORAL SEX)

1. **Did you ever have oral sex (explanation using Dutch commonly used words: ‘pijpen’ or ‘beffen’) ?**

1 □ no

2 □ yes

1. **With how many different persons did you have oral sex in the past 12 months?**

with ……. different persons

(ANAL SEX)

1. **Did you ever have anal sex? (explanation using Dutch commonly used word: ‘kont neuken’)**

1 □ no

2 □ yes

1. **With how many different persons did you have anal sex in your life?**

with ……. different persons

1. **Did you have anal sex with a steady partner in the past 12 months?**

□ yes

□ no

1. **With how many steady partners did you have anal sex in the past 12 months?**

with ……. steady partners

1. **How often did you use condoms during anal sex with a steady partner in the past 12 months?**

□ never

□ most of the times not

□ sometimes yes, sometimes not

□ most of the times

□ always

1. **Did you have anal sex with a casual partner in the past 12 months?**

□ yes

□ no

1. **With how many casual partners did you have anal sex in the past 12 months?**

with ……. steady partners

1. **How often did you use condoms during anal sex with a casual partner in the past 12 months?**

□ never

□ most of the times not

□ sometimes yes, sometimes not

□ most of the times

□ always

# Part 5: Experiences with STI

What are STI?

STI: Sexual transmittable infections (venereal diseases). Diseases that you may get if you have unsafe sex. Examples are Chlamydia, Gonorrhea, Syphilis, Herpes or HIV.

HIV: the virus that causes Aids.

1. **Were you ever tested for STI (excluding HIV) ?**

1 □ No

2 □ yes, once

3 □ yes, more than once

1. **When was the last time?**

□ less than one month ago

□ between 1 and 6 months ago

□ between 6 and 12 months ago

□ more than 12 months ago

1. **What was the test result?**

1 □ I had no STI

2 □ I did have an STI: □ Chlamydia

□ Genital wrats

□ Herpes

□ Gonnorroe

□ Syfilis

□ something else: …………………………………….

□ I don’t know

□ results are not yet known

1. **Where did you do the STI test?**

□ At my general practitioner

□ At the STI-clinc at the hospital

□ At the CASA (Centre for contraceptives, sexuality en abortion)

□ At a municipal health service

□ somewhere else:

1. **Were you ever tested for HIV?**

1 □ No

2 □ yes, once

3 □ yes, more than once

1. **When was the last time?**

□ less than one month ago

□ between 1 and 6 months ago

□ between 6 and 12 months ago

□ more than 12 months ago

1. **Where did you do the STI test?**

□ At my general practitioner

□ At the STI-clinic at the hospital

□ At the CASA (Centre for contraceptives, sexuality en abortion)

□ At a municipal health service

□ somewhere else:

1. **What was the test result?**

1 □ I was **not** infected with HIV (sero-negative)

2 □ I was infected with HIV (sero-positive)

1. **What are your plans with respect to testing?**

|  | | **certainly yes** | **probably yes** | **maybe yes, maybe no** | **probably not** | **certainly not** |
| --- | --- | --- | --- | --- | --- | --- |
| **1** | I intent to take a test for HIV | □ | □ | □ | □ | □ |
| **2** | I intent to take a test for STI | □ | □ | □ | □ | □ |
| **3** | I am planning to take a test for HIV. | □ | □ | □ | □ | □ |
| **4** | I am planning to take a test for STI. | □ | □ | □ | □ | □ |

1. **Imagine, you had sex with someone without a condom. How do you think about going for an STI test?**

I think it is

|  | □ very unpleasant | □ unpleasant | □ not pleasant/not unpleasant | □ unpleasant | □ very unpleasant |
| --- | --- | --- | --- | --- | --- |

I think it is

:

|  | □ very wise | □ wise | □ not wise/ not unwise | □ unwise | □ very unwise |
| --- | --- | --- | --- | --- | --- |

1. **Imagine the following situation:**

**You have had a relationship in the past, and you have had sex without a condom. Now, you have new girlfriend/boyfriend and you want to have sex without a condom. How do you think about going for an STI test first?**

I think it is

|  | □ very unpleasant | □ unpleasant | □ not pleasant/not unpleasant | □ unpleasant | □ very unpleasant |
| --- | --- | --- | --- | --- | --- |

I think it is

|  | □ very wise | □ wise | □ not wise/ not unwise | □ unwise | □ very unwise |
| --- | --- | --- | --- | --- | --- |

1. **Imagine, you had sex with someone without a condom. Would you go for an STI test?**

□ certainly not

□ probably not

□ maybe no / maybe yes

□ probably yes

□ certainly yes

1. **Imagine the following situation:**

**You have had a relationship in the past, and you have had sex without a condom. Now, you have a new girlfriend/boyfriend and you want to have sex without a condom. Would you go first go for an STI test?**

□ certainly not

□ probably not

□ maybe no / maybe yes

□ probably yes

□ certainly yes

1. **How likely do you think it is that you get an STI if you have sexual intercourse with.............**

|  |  | **not likely at all** | **quite unlikely** | **not unlikely, not likely** | **quite likely** | **very likely** |
| --- | --- | --- | --- | --- | --- | --- |
| 1 | someone you do not know very well | □ | □ | □ | □ | □ |
| 3 | someone you met on holiday | □ | □ | □ | □ | □ |
| 4 | someone you met through friends | □ | □ | □ | □ | □ |
| 5 | someone you have been in a relationship with | □ | □ | □ | □ | □ |
| 6 | someone you met on the internet | □ | □ | □ | □ | □ |

1. **If you want to take an STI test, is it important to you, that...............**

|  |  | **very important** | **important** | **not unimportant/ no important** | **unimportant** | **very unimportant** |
| --- | --- | --- | --- | --- | --- | --- |
| **1** | … you can see a doctor or nurse that does not know you? | □ | □ | □ | □ | □ |
| **2** | … the test site is nearby? | □ | □ | □ | □ | □ |
| **3** | … you don’t have to give your real name? | □ | □ | □ | □ | □ |
| **4** | … you are treated with confidentiality? | □ | □ | □ | □ | □ |
| **5** | … you don’t have to wait longer than a few days for the results? | □ | □ | □ | □ | □ |
| **6** | … you can choose how you receive the test results? | □ | □ | □ | □ | □ |
| **7** | … it is free of charge? | □ | □ | □ | □ | □ |
| **8** | … opening hours are in the evening? | □ | □ | □ | □ | □ |
| **9** | … opening hours are during the day? | □ | □ | □ | □ | □ |

1. **Maybe you have never taken an STI test, and maybe you have no plans to do so.**

**But try to imagine how you think about the following:**

| **“If I take an STI test …”** | | **completely agree** | **agree** | **not agree/ not disagree** | **disagree** | **completely disagree** |
| --- | --- | --- | --- | --- | --- | --- |
| **1** | I don’t have to be uncertain anymore | □ | □ | □ | □ | □ |
| **2** | and have to wait a while for the results, I will get totally stressed out’ | □ | □ | □ | □ | □ |
| **3** | I am afraid that they tell me I have an STI’ | □ | □ | □ | □ | □ |
| **4** | I can prevent to infect others | □ | □ | □ | □ | □ |
| **5** | I can get treated for STI before it is too late. | □ | □ | □ | □ | □ |
| **6** | I don’t have to worry anymore about things that happened in the past | □ | □ | □ | □ | □ |
| **7** | I am afraid that a positive test result would turn my life upside down | □ | □ | □ | □ | □ |
| **8** | I don’t like to pay a lot for it | □ | □ | □ | □ | □ |
| **9** | it will be a good moment to always have safe sex again | □ | □ | □ | □ | □ |
| **10** | I take responsibility for my own health | □ | □ | □ | □ | □ |
| **11** | I take responsibility for the health of sex partner(s) | □ | □ | □ | □ | □ |
| **12** | I know where I stand | □ | □ | □ | □ | □ |
| **13** | I can have sex without a condom | □ | □ | □ | □ | □ |
| **14** | I show my partner that I am serious | □ | □ | □ | □ | □ |
| **15** | it means that I don’t trust my partner enough | □ | □ | □ | □ | □ |
| **16** | I do not dare to go to my GP; I might have to show my private parts | □ | □ | □ | □ | □ |
| **17** | I think it is painful and embarrassing | □ | □ | □ | □ | □ |

1. If you had an STI, how would you feel?

|  |  | **certainly yes** | **probably yes** | **maybe yes, maybe no** | **probably not** | **certainly not** |
| --- | --- | --- | --- | --- | --- | --- |
| 1 | I would feel ashamed | □ | □ | □ | □ | □ |
| 2 | I would feel embarressed | □ | □ | □ | □ | □ |
| 3 | I would feel guilty | □ | □ | □ | □ | □ |
| 4 | I would be afraid | □ | □ | □ | □ | □ |
| 5 | I would feel disappointed in myself | □ | □ | □ | □ |  |

1. If you had an STI, do you think that ........

|  |  | **certainly yes** | **probably yes** | **maybe yes, maybe no** | **probably not** | **certainly not** |
| --- | --- | --- | --- | --- | --- | --- |
| 1 | people would avoid you? | □ | □ | □ | □ | □ |
| 2 | people would think you were unclean? | □ | □ | □ | □ | □ |
| 3 | other people would think badly of you? | □ | □ | □ | □ | □ |
| 4 | other people would not want to be friends with you? | □ | □ | □ | □ | □ |
| 5 | other people would be disgusted by you? | □ | □ | □ | □ | □ |
| 6 | other people would be uncomfortable around you? | □ | □ | □ | □ | □ |

1. **Imagine, you want to do an STI test**

**How easy of difficult do you think that it is to .....**

|  |  | **very difficult** | **rather difficult** | **not difficult/not easy** | **quite easy** | **very easy** |
| --- | --- | --- | --- | --- | --- | --- |
| 1 | find information about STI testing? | □ | □ | □ | □ | □ |
| 2 | to make an appointment for an STI test? | □ | □ | □ | □ | □ |
| 3 | go to the test-site (for example to the STI-clinic or to your general practitioner) ? | □ | □ | □ | □ | □ |
| 4 | discuss your sexual behavior with a nurse of doctor? | □ | □ | □ | □ | □ |
| 5 | have the test performed? | □ | □ | □ | □ | □ |
| 6 | wait a few days for the results? | □ | □ | □ | □ | □ |
| 7 | receive the test results? | □ | □ | □ | □ | □ |
| 8 | have a conversation with the nurse or doctor when you receive the result? | □ | □ | □ | □ | □ |

1. **[only if respondent has steady partner] What does your partner think**?

|  |  | **certainly yes** | **probably yes** | **maybe yes, maybe no** | **probably not** | **certainly not** | **no idea what my partner thinks/does** |
| --- | --- | --- | --- | --- | --- | --- | --- |
| **1** | Does your partner think that you should take an STI test? | □ | □ | □ | □ | □ | □ |
| **2** | Is your partner tested for STI? | □ | □ | □ | □ | □ |  |
|  |  |  |  |  |  |  |  |
| **3** | Do you think your partner will support you if you take an STI test? | □ | □ | □ | □ | □ | □ |
| **4** | Do you think your partner will support after an unfavorable test result? | □ | □ | □ | □ | □ | □ |

1. **Wat about your friends?**

|  |  | **none of them** | **most of them not** | **half of them** | **most of them** | **all of them** | **I have no idea** | **not applicable** |
| --- | --- | --- | --- | --- | --- | --- | --- | --- |
| **1** | Do your friends think that you should take an STI test before you have unprotected sex? | □ | □ | □ | □ | □ | □ | □ |
| **2** | You have a new boyfriend/girl friend and you want to have sex with her/him without a condom. Do your friends think that you should first take an STI test? | □ | □ | □ | □ | □ | □ | □ |
| **3** | My friends are tested for STI | □ | □ | □ | □ | □ | □ | □ |
| **4** | If I take an STI test, my friends will support me | □ | □ | □ | □ | □ | □ | □ |
| **5** | If I receive unfavorable test results, my friends will support me | □ | □ | □ | □ | □ | □ | □ |

1. **What do you parents (or step- or foster- parents) think?**

|  |  | **certainly yes** | **probably yes** | **maybe yes, maybe no** | **probably not** | **certainly not** | **no idea what they think** | **not applicable** |
| --- | --- | --- | --- | --- | --- | --- | --- | --- |
| **1** | Do your parents think that you should take an STI test before you have unprotected sex? | □ | □ | □ | □ | □ | □ | □ |
| **2** | You have a new boyfriend/girl friend and you want to have sex with her/him without a condom. Do your parents think that you should first take an STI test | □ | □ | □ | □ | □ | □ | □ |
| **3** | If I take an STI test, my parents will support me | □ | □ | □ | □ | □ | □ | □ |
| **4** | If I receive unfavorable test results, my parents will support me | □ | □ | □ | □ | □ | □ | □ |
